# An Integrated Text Mining Approach for Discovering Pharmacological Effects, Drug Combinations, and Repurposing Opportunities of ACE Inhibitors

**DOI:** 10.3390/ijms27042044

**Published:** 2026-02-22

**Authors:** Nadezhda Yu. Biziukova, Polina I. Savosina, Dmitry S. Druzhilovskiy, Olga A. Tarasova, Vladimir V. Poroikov

**Affiliations:** Institute of Biomedical Chemistry, 10, Pogodinskaya Street, 119121 Moscow, Russia; polina.savosina@ibmc.msk.ru (P.I.S.); dmitry.druzhilovsky@ibmc.msk.ru (D.S.D.); olga.a.tarasova@gmail.com (O.A.T.)

**Keywords:** hypertension, drugs, ACE inhibitors, text mining, pharmacological action

## Abstract

The rapidly expanding body of biomedical literature encompasses a wealth of information concerning the pharmacological effects, mechanisms of action, adverse reactions, and repurposing potential of small-molecule therapeutics. Nevertheless, the systematic extraction and integration of this knowledge continue to pose substantial challenges. In this study, we propose an integrated text-mining framework for the automated extraction and structured representation of information on the biological activities of low-molecular-weight compounds, exemplified by angiotensin-converting enzyme (ACE) inhibitors as a representative pharmacological class. A corpus comprising over 20,000 PubMed titles and abstracts reporting in vitro, in vivo, and clinical investigations of ACE inhibitors was assembled. Chemical compounds, proteins/genes, and diseases were recognized using a previously developed named entity recognition model based on conditional random fields. Entity-level associations were extracted at the sentence level through a rule-based approach employing manually curated pattern phrases, followed by normalization via automated queries to PubChem, UniProt, and the Human Disease Ontology. The proposed methodology facilitated the extraction of approximately 22,000 unique and normalized associations encompassing drug-target, drug-disease, and drug-drug relationships. In addition to confirming well-established therapeutic effects and clinically recognized drug combinations, the analysis identified underexplored pharmacological activities of ACE inhibitors, including antineoplastic, antifibrotic, and neuropsychiatric properties, along with mechanistic associations involving matrix metalloproteinases and neurotrophic signaling pathways. Collectively, these findings underscore the potential of automated literature mining to advance systematic knowledge integration and data-driven hypothesis generation in the contexts of drug repurposing and safety evaluation.

## 1. Introduction

Information on the biological activity of small-molecule compounds is essential for drug discovery, safety assessment, and the development of novel therapeutic strategies. In addition to primary therapeutic effects, such information includes adverse drug reactions, potential drug–drug interactions, and opportunities for drug repurposing. Systematic aggregation of these data is particularly important for computational approaches, including quantitative structure–activity relationship modeling and in silico safety prediction, which rely on comprehensive and consistent representations of known biological effects [1,2].

Scientific publications remain the principal source of comprehensive information regarding the pharmacological effects and mechanisms of action of therapeutic agents. Nevertheless, the rapid expansion of biomedical literature has yielded tens of millions of articles encompassing a wide array of experimental methodologies, biological models, and clinical contexts. The magnitude and heterogeneity of these data render manual analysis and systematic integration of published findings increasingly infeasible, even within narrowly defined pharmacological classes.

Text mining approaches offer a promising solution for automated extraction and structuring of relevant information from large collections of scientific texts. Numerous methods and tools have been developed for specific subtasks, such as named entity recognition, relation extraction, or literature-based discovery [3,4]. Large-scale initiatives have also focused on reconstructing molecular mechanisms from text-mined statements [5]. Nevertheless, most existing approaches address isolated aspects of text analysis or focus predominantly on molecular interactions, while comprehensive pipelines for extracting pharmacologically relevant knowledge from user-defined literature collections remain limited.

In this study, we propose an integrated text-mining framework designed to extract and structure information on the biological activities of low-molecular-weight compounds. The framework incorporates automated literature retrieval, recognition of chemical and biological entities, and extraction of biologically relevant associations through curated linguistic patterns. As a case study, the proposed pipeline is applied to angiotensin-converting enzyme (ACE) inhibitors, a well-characterized pharmacological class exhibiting pleiotropic effects that extend beyond their established therapeutic use in cardiovascular disorders. The primary objective of this work is to illustrate the potential of automated literature mining to facilitate systematic knowledge consolidation, uncover underexplored pharmacological properties, and support hypothesis generation for drug repurposing and safety evaluation.

## 2. Results

### 2.1. Characteristics of the Text Corpus

As a result of automated retrieval and filtering, a corpus comprising 24,294 PubMed records was assembled, with each row corresponding to a unique PubMed identifier (PMID). The publication years of the collected records ranged from 1958 to 2026, covering more than six decades of biomedical research.

Titles were available for >99.9% of records, while abstracts were present for 93.8% of publications. Missing abstracts were observed in 6.19% of records, primarily corresponding to older publications. MeSH terms were available for 99.1% of records. Journal names, publication years, study type annotations, and drug labels were present for all records.

Table 1 summarizes the distribution of texts across individual angiotensin-converting enzyme inhibitors and study types. The corpus was dominated by publications describing enalapril and captopril, which together accounted for 20,220 texts, corresponding to 86.81% of all documents in the corpus. Enalapril was the most frequently mentioned compound, with 10,635 texts (45.66% of the corpus), followed by captopril with 9585 texts (41.15%).

A second tier of compounds was represented by benazepril (4091 texts; 17.56%), ramipril (1895 texts; 8.14%), lisinopril (1885 texts; 8.09%), and perindopril (1512 texts; 6.49%). All remaining ACE inhibitors individually accounted for less than 6% of the corpus, with alacepril being the least represented compound (69 texts; 0.3%).

Across the corpus, in vivo studies constituted the largest proportion of texts for most compounds. For example, in vivo publications accounted for 56.73% of captopril-related texts and 57.15% of enalaprilat-related texts. In contrast, clinical studies were predominant for several compounds, including benazepril (50.28% clinical texts), trandolapril (52.38%), spirapril (58.33%), and moexipril (44.44%).

In vitro studies represented the smallest fraction of publications for the majority of compounds. The proportion of in vitro texts ranged from 9.26% for spirapril to 30.39% for enalaprilat. For widely studied compounds such as enalapril and captopril, in vitro publications accounted for 16.30% and 25.09% of texts, respectively. Overall, the corpus exhibits a strong imbalance in both compound representation and study type distribution, with a small number of ACE inhibitors dominating the literature and with in vivo and clinical studies substantially outnumbering in vitro investigations.

### 2.2. Rule-Based Association Extraction: Structure and Outcomes

#### 2.2.1. Semantic Groups of Pattern Phrases

Application of the rule-based extraction framework resulted in a structured inventory of pattern phrases reflecting the dominant linguistic strategies used in the biomedical literature to describe pharmacologically relevant relationships. All patterns were organized into three semantic groups corresponding to the main types of associations investigated in this study: mechanisms of action, pharmacological effects, and drug co-administration or co-prescription.

The Mechanisms group constituted the largest and most diverse category, comprising 42 pattern subgroups and a total of 248 lexical patterns (Table 2). These patterns capture statements describing molecular and cellular interactions between angiotensin-converting enzyme inhibitors and biological targets, including genes, proteins, and signaling pathways. Subgroups within this category varied in indicative strength, with strong patterns typically corresponding to explicit mechanistic statements (e.g., “inhibits expression of”, “activates”), medium-strength patterns reflecting modulatory or associative relationships (e.g., “is involved in”, “modulates activity of”), and weak patterns representing more indirect or context-dependent descriptions frequently encountered in mechanistic discussions.

The Effects group included 33 subgroups and 167 lexical patterns and was used to capture associations between drugs and diseases or phenotypic outcomes. Despite its smaller size compared to the Mechanisms group, this category exhibited comparable internal structure, with a median of six lexical patterns per subgroup (Table 2). Strong patterns in this group predominantly corresponded to explicit statements of therapeutic efficacy or adverse outcomes (e.g., “is effective in the treatment of”, “induces”, “causes”), whereas medium- and weak-strength patterns reflected more heterogeneous clinical and experimental formulations, such as improvement, attenuation, or associative links between drug exposure and disease-related outcomes.

The Co-administration group was intentionally compact and consisted of three subgroups encompassing 13 lexical patterns. This limited size reflects the relatively standardized language used to describe drug combinations and joint therapeutic regimens in scientific texts. Patterns in this group were primarily of strong or medium indicative strength and included formulations such as “in combination with”, “co-administered with”, and “added to standard therapy”. Weak patterns were rarely observed in this category, consistent with the tendency of authors to describe drug combinations using explicit and well-defined expressions.

#### 2.2.2. Sentence-Level Associations Extracted from the Text Corpus

Application of the rule-based pattern inventory to the text corpus resulted in the extraction of a large set of sentence-level associations describing mechanisms of action, pharmacological effects, and drug co-prescription. At the initial stage, associations were identified without restricting the participating chemical entities to angiotensin-converting enzyme inhibitors, which allowed the detection of all pattern-consistent relations present in the texts, including those involving other small-molecule compounds mentioned in the same context.

To focus the subsequent analysis on the target pharmacological class, the extracted associations were filtered according to the presence of at least one angiotensin-converting enzyme inhibitor. This filtering step substantially reduced the dataset, retaining 6722 sentence-level associations out of 273,679 initially extracted relations, while excluding associations that did not involve ACE inhibitors. All results described below refer exclusively to this filtered set of sentence-level associations.

Entity normalization was applied to the filtered associations to enable consistent aggregation and analysis. For the majority of extracted relations (almost 93%), both participating entities were successfully normalized to external reference identifiers. A smaller fraction of associations contained only one normalized entity, while no associations remained in which neither entity could be normalized. This outcome indicates that the extracted relations predominantly involve well-defined chemical and biological entities and are suitable for subsequent integration and aggregation.

#### 2.2.3. Aggregation of Associations and Network Representation

Following the filtering of sentence-level associations to retain only unique relations extracted from the corpus for ACE inhibitors, the resulting dataset comprised a total of 22,395 aggregated associations (available at Supplementary Materials). Among them, approximately 11% corresponded to negative statements, indicating experimentally tested but unsupported or explicitly excluded relationships, while the remaining associations reflected affirmative evidence. On average, each aggregated association was supported by 2.46 independent PubMed records, highlighting the recurrent nature of many extracted relations across the literature.

The extracted associations were unevenly distributed across the three semantic groups. The largest number of associations was observed for Mechanisms (10,659 associations) and Effects (10,412 associations), which together accounted for the vast majority of the dataset. In contrast, the Co-prescription group was substantially smaller, comprising 1324 associations. This distribution reflects both the research focus of the literature on molecular mechanisms and pharmacological outcomes of ACE inhibitors and the more limited scope of explicitly described drug–drug combinations.

A more detailed view of the extracted associations is provided by the distribution of relation types within each semantic group (Table 3). The most frequent relation type overall was Mechanisms-Inhibit, comprising 2086 associations, consistent with the central role of inhibitory processes in studies of ACE inhibitor mechanisms of action. Within the Effects group, relation types such as Effect, Treatment, and Patient were most prevalent, reflecting the frequent discussion of ACE inhibitors as components of therapeutic regimens and their impact on patient-related outcomes. The Co-prescription group was dominated by relation types explicitly describing joint drug usage, including Cooccurrence and Coadministered_with, which together accounted for the majority of associations in this category. Other relation types observed among the top-ranked associations capture modulatory, preventive, or attenuating effects, illustrating the diversity of mechanistic and phenotypic contexts represented in the literature.

Analysis of signal strength distribution across the aggregated associations further revealed group-specific patterns (Table 4). In the Mechanisms group, most associations were extracted using medium-strength patterns, followed by strong patterns, while weak patterns were relatively infrequent. This suggests that mechanistic relationships are often described using formulations that imply modulation or involvement rather than explicit causality. In contrast, the Effects group exhibited a more balanced distribution between medium- and weak-strength patterns, reflecting the heterogeneous and context-dependent language used to describe pharmacological outcomes in experimental and clinical studies. Associations in the Co-prescription group were predominantly extracted using strong or weak patterns, consistent with the relatively explicit and standardized phrasing employed to describe drug combinations.

**Figure 1:**
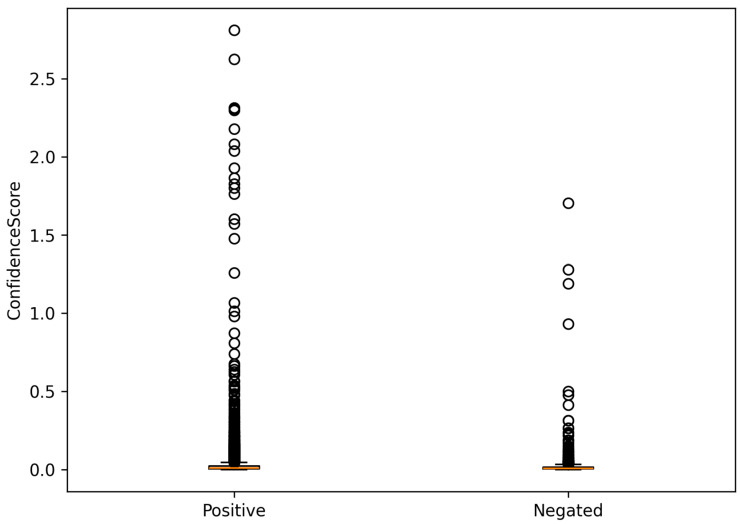
Distribution of confidence scores for positive and negated associations.

To further assess differences between affirmative and negated associations, the distribution of confidence scores was examined using box-and-whisker plots (Figure 1). Positive associations exhibited higher median confidence scores and a broader interquartile range, with a pronounced upper tail corresponding to highly supported and recurrent relationships. In contrast, negated associations were characterized by lower confidence scores and a more compact distribution, indicating weaker and less consistent evidence.

This separation supports the decision to treat positive and negated associations as distinct categories in subsequent analyses and confirms the ability of the confidence scoring framework to capture meaningful differences in evidential support.

To further examine how different study types contribute to the structure of the aggregated association set, we performed a comparative analysis of associations supported exclusively by in vitro studies and those supported by in vivo or clinical studies. Associations derived from in vivo or clinical publications substantially dominated the dataset, accounting for 21,232 aggregated associations compared to 1163 associations supported exclusively by in vitro evidence. Associations supported by in vivo or clinical studies exhibited a higher density of evidential support, with an average of 3.92 association mentions per publication, compared to 2.78 for in vitro-only studies.

Despite the lower overall contribution, in vitro-only publications more frequently introduced unique associations, with a higher number of unique associations per publication (2.70 versus 1.54 for in vivo or clinical studies). However, temporal analysis revealed that such associations were typically short-lived: the majority of in vitro-only associations were observed in a single publication year, with a mean temporal span of 0.12 years. In contrast, associations supported by in vivo or clinical studies demonstrated substantially greater temporal persistence, with a mean span of 3.40 years and repeated support across multiple years.

**Figure 2:**
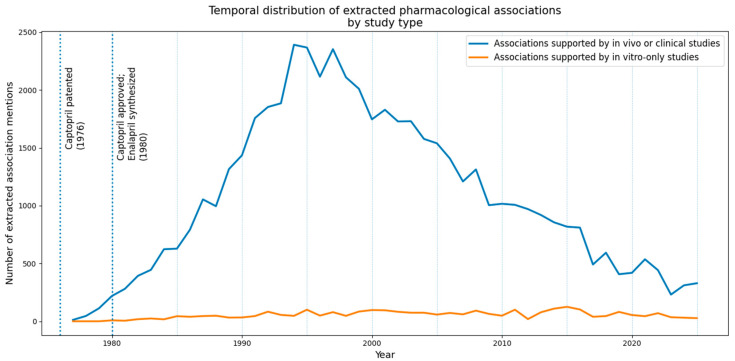
Temporal distribution of extracted pharmacological association mentions by study type.

These differences suggest that temporal persistence provides a useful criterion for distinguishing exploratory or potentially unstable associations from relations that undergo repeated confirmation across independent studies for the evaluated set of text annotations. While in vitro studies contribute mechanistically focused and potentially novel associations, the aggregated association networks are predominantly shaped by in vivo and clinical literature, which provides denser and more temporally stable evidential support. The temporal dynamics of these differences are illustrated in Figure 2, which shows the annual distribution of extracted association mentions stratified by study type.

Across the entire time span, associations supported by in vivo or clinical studies consistently outnumber those supported exclusively by in vitro evidence. Notably, a sharp increase in the number of extracted associations is observed following key milestones in the development of ACE inhibitors, including the patenting of captopril in 1976 and its clinical approval together with the synthesis of enalapril in 1980. This growth reflects not only increased research activity but also the repeated confirmation and consolidation of pharmacological knowledge in later-stage studies. In contrast, associations derived exclusively from in vitro studies remain comparatively limited throughout the period, forming a persistent but substantially smaller layer of the aggregated association set.

## 3. Discussion

Although the present study focuses on a single, well-characterized pharmacological class, the proposed framework was developed with adaptability in mind. In practice, named entity recognition for drugs is based on machine learning/artificial intelligence methods, and the inventory of linguistic patterns used for association extraction is not intended to be static or universally fixed. For different biomedical tasks or drug classes, the pattern set may require task-specific adjustment, including both extension and restriction [6,7]. At the same time, our experience across multiple text-mining applications indicates that a core subset of patterns capturing fundamental pharmacological relations (e.g., inhibition, activation, therapeutic use) remains stable across domains, whereas more specialized patterns tend to be context-dependent. This design supports both transferability and interpretability of the approach and provides a basis for its application beyond the specific case study considered here.

In the Section 2,we focused on providing a quantitative overview of the analyzed corpus and the associations extracted from the literature, without delving into the substantive interpretation of individual relationships. The primary aim of the present study was not to discover novel mechanisms or effects for a poorly characterized drug class, but rather to demonstrate the applicability of the proposed text-mining and aggregation framework for organizing and summarizing information available in biomedical texts. Angiotensin-converting enzyme inhibitors, representing a well-studied group of antihypertensive drugs, offer a suitable case for illustrating how heterogeneous textual evidence can be consolidated and subsequently interpreted in a structured manner.

Given the large number of extracted associations, network construction was performed using multiple confidence score thresholds, progressively restricting the graphs to relations with higher estimated reliability. This threshold-based filtering made it possible to balance network completeness against visual interpretability. As shown in Table 5, the number of retained associations decreases substantially with increasing confidence score thresholds, with particularly pronounced reductions observed for the effects and co-prescription groups. For inclusion in the main text, we selected only those networks for which representation as a static, non-scalable figure is feasible, that is, graphs with a limited number of nodes and edges that can be meaningfully inspected without interactive exploration. These networks are highlighted in Table 5. Networks constructed at lower confidence thresholds, as well as more densely connected graphs that exceed the limits of static visualization, are provided in the Supplementary Materials.

### 3.1. Associations of ACE Inhibitors Revealed by Text Mining

**Figure 3:**
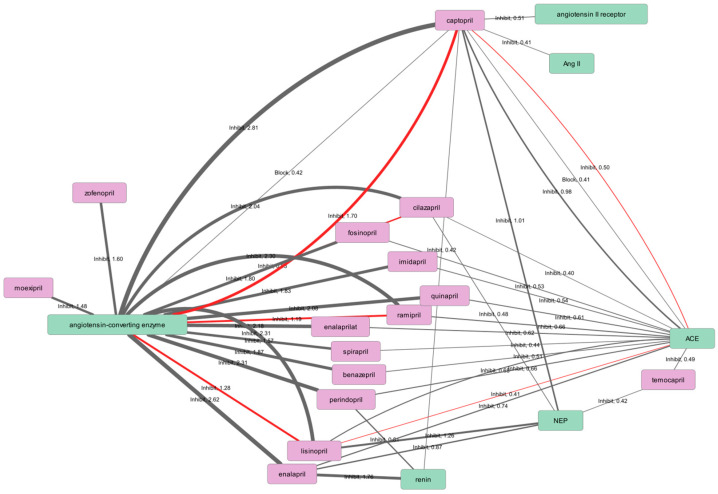
Mechanistic association network for angiotensin-converting enzyme inhibitors constructed at a confidence score threshold ≥ 0.5. Node colors distinguish entity types (proteins/genes in green and chemical compounds in pink). Edge thickness reflects the confidence score of the extracted associations, while red edges indicate negated associations.

The mechanistic network constructed at a confidence score threshold of ≥0.5 provides an interpretable summary of how angiotensin-converting enzyme inhibitors are described in sentence-level mechanistic statements across the analyzed corpus (Figure 3). At this threshold, the network is dominated by the expected, canonical pharmacological mechanism: the majority of ACE inhibitors are linked to the angiotensin-converting enzyme through inhibitory relations. This network configuration, in which multiple drugs converge on a single principal molecular target, is consistent with the fact that ACE inhibitors constitute a well-established therapeutic class for which the literature repeatedly emphasizes a shared primary mode of action. In this sense, the network serves as a validation of the rule-based extraction strategy: the most prominent structure is not an artifact of aggregation, but rather a reflection of the central mechanistic emphasis in biomedical texts.

Compounds such as captopril and enalapril/enalaprilat appear more central in the network, forming a greater number of mechanistic connections. This centrality primarily reflects their role as early, extensively studied reference compounds rather than an intrinsic dominance within the ACE inhibitor class. Captopril was the first angiotensin-converting enzyme inhibitor approved for clinical use, receiving U.S. FDA approval in 1981 and subsequently becoming a widely studied reference compound in both experimental and clinical research.

Enalapril, which followed as one of the earliest nonsulfhydryl ACE inhibitors, was introduced to the market in 1981-1982 as well, shortly after captopril, and also became a commonly used reference substance in antihypertensive studies. As a result, the aggregated association networks are influenced by the uneven distribution of research attention across individual drugs, and less frequently studied ACE inhibitors may appear to have fewer reported associations—not because such effects or interactions are absent, but because they are less densely represented in the literature.

Although the canonical ACE-inhibitor relations dominate the network, additional nodes associated with the renin-angiotensin system (RAS) and adjacent pathways also appear, including concepts such as renin, angiotensin II (Ang II), angiotensin II receptors, and neprilysin (NEP). In a well-studied pharmacological system like RAS, mechanistic descriptions in the literature often move between direct molecular inhibition (ACE), downstream effector changes (Ang II levels), receptor-mediated signaling, and system-level modulation. Sentence-level pattern matching can therefore capture associations that are mechanistically contextual rather than direct. For example, Ang II is frequently mentioned in experimental designs as a perturbation (Ang II stimulation) [8], a downstream readout (changes in Ang II concentration) [9,10,11], or a rescue condition (reversal of effects by exogenous Ang II) [12].

In such settings, co-occurrence with ACE inhibitors and a mechanistic trigger phrase can yield an extracted relation between the inhibitor and Ang II even when the underlying biology is indirect (i.e., mediated by ACE inhibition rather than direct inhibition of Ang II itself). A similar logic applies to angiotensin II receptors: ACE inhibitors and angiotensin receptor blockers are routinely discussed side-by-side as alternative or complementary approaches to modulating RAS [13,14,15,16], and the textual proximity of ACE inhibitors to receptor terminology can produce extracted edges that reflect comparative framing rather than receptor antagonism by ACE inhibitors. Accordingly, both the prominence of specific drugs and the apparent balance between positive and negative associations should be interpreted in light of underlying literature biases rather than as a direct representation of class-wide pharmacological effects. Thus, edges involving Ang II or its receptors can be understood as signatures of system-level mechanistic context in the corpus, which becomes visible once the associations are aggregated and represented as a network.

The observation that multiple relation types connect the same drug–entity pair further supports the interpretation that these non-canonical edges predominantly reflect contextual mechanistic framing. Even within the mechanistic group, relations are not limited to a single predicate such as “inhibit”, but include additional lexical categories such as “block,” and (in the aggregated tables) terms indicating increased or reduced levels. This diversity is expected in a literature that describes causal chains and experimental interventions rather than only direct binding or enzymatic inhibition. At the same time, it creates a conceptual distinction that should be maintained in interpretation: “block” and “inhibit” are not equivalent across all contexts, and “block” may refer to pathway-level suppression, functional blockade, or downstream signaling attenuation rather than direct enzyme inhibition. In the present framework, these relation types correspond to curated pattern groups and therefore capture the surface semantics of the sentence. Consequently, relation labels are best interpreted as a structured summary of how a mechanistic claim is phrased in text, which can then guide targeted manual review when a direct mechanistic interpretation is desired. Importantly, the associations extracted by the proposed framework should not be interpreted as direct evidence of causal relationships. In many cases, they reflect observed experimental outcomes, contextual mechanistic descriptions, or correlations reported in the literature rather than proven cause-effect links. The framework captures how relations are described in text, not the strength or direction of biological causality. Accordingly, different relation types and confidence scores indicate linguistic and evidential support rather than mechanistic certainty, and the resulting networks are best viewed as structured maps of reported knowledge that can guide targeted expert review and hypothesis generation.

Another notable feature of the network is the persistence of negated edges (colored red) even under a relatively strict confidence threshold. This highlights an important characteristic of biomedical discourse: mechanistic claims are often stated in a conditional, comparative, or negative form, especially in experimental contexts where “no effect” findings, dose dependencies, tissue-specific outcomes, or contradictory results across models are discussed [17]. Retaining polarity at the aggregation stage prevents these statements from being silently merged into positive evidence and allows the network to represent not only the dominant mechanistic narrative but also the presence of negative or limiting statements. In practice, negated edges can mark contexts where an expected mechanistic effect was tested and not observed, where a hypothesized mechanism was explicitly ruled out, or where the statement pertains to a comparator (e.g., an angiotensin receptor blocker) rather than the ACE inhibitor itself. Therefore, polarity annotation is not merely a technical feature of the extraction pipeline; it materially affects interpretability of aggregated mechanistic knowledge.

At the same time, the mechanistic network provides a useful lens on limitations of automated normalization, particularly for abbreviations and short forms. In the present graph, the label “ACE” does not correspond to the angiotensin-converting enzyme target; instead, it was erroneously normalized to an unrelated protein (Protachykinin-1). Similarly, the node labeled “Ang II” was normalized to an unrelated identifier (proliferating cell nuclear antigen). These cases illustrate abbreviation ambiguity and underscore that normalization accuracy can become the main determinant of biological plausibility once results are aggregated into networks. In practical terms, such errors can create artificial “duplicate targets,” fragment evidence across nodes that should represent the same concept, and introduce spurious links that appear mechanistic but in fact arise from mislabeled identifiers. This does not invalidate the sentence-level extraction itself—because the underlying text evidence can still be correct—but it does indicate that high-quality network interpretation requires a post-extraction harmonization stage, including abbreviation disambiguation and synonym merging for high-frequency concepts such as ACE and Ang II. The fact that these normalization artifacts become visually salient in the network representation is itself valuable: the graph acts as a diagnostic tool that highlights which entities require curation before downstream biological interpretation.

Because the confidence score used in this work is a composite measure and is not normalized to the [0, 1] interval, its numerical values should be interpreted relative to the scoring scheme rather than as absolute probabilities. A threshold of ≥0.5 thus represents a pragmatic cutoff on the internal confidence scale adopted here, aimed at retaining relations supported by stronger textual and normalization evidence while filtering low-confidence co-occurrences. This is particularly relevant when interpreting edge thickness, which is proportional to the score: thickness reflects relative ranking within the chosen confidence range rather than a probabilistic measure of truth.

### 3.2. Low-Frequency Associations and Their Interpretative Significance

**Figure 4:**
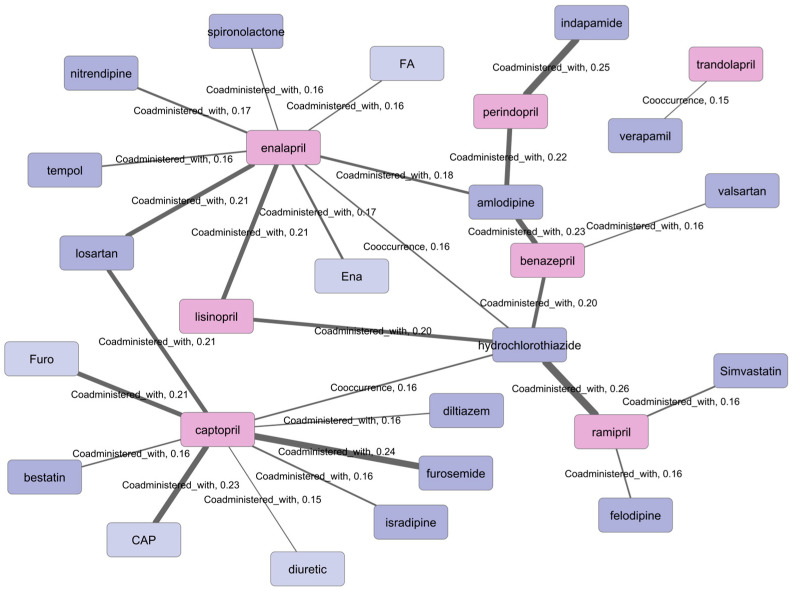
Co-prescription network involving angiotensin-converting enzyme inhibitors constructed at a confidence score threshold ≥ 0.15. Node colors indicate drug classes: pink nodes represent ACE inhibitors, while purple nodes denote other drugs co-prescribed in combination therapies. Lighter node shades indicate entities that were not normalized. Edge thickness reflects the confidence score of the extracted associations.

The most frequently represented combinations among the associations extracted from the literature involve pairs of ACE inhibitors and antihypertensive drugs with alternative mechanisms of action (Figure 4). This observation is not unexpected, as a substantial proportion of the analyzed corpus (over 30%) consists of clinical trial reports, in which ACE inhibitors are typically discussed as components of well-established combination regimens for the treatment of cardiovascular diseases [18].

Less frequently represented in the literature are combinations of ACE inhibitors with diuretics, statins, hypoglycemic agents, antineoplastic, and anti-inflammatory compounds. Among these, examples of drug pairs exhibiting synergistic effects can be found. For instance, the combination of lisinopril and empagliflozin demonstrated superior efficacy compared with monotherapy in mouse models of diabetic nephropathy by slowing the progression of structural alterations in renal tissue, resulting in an enhanced hypoglycemic effect [19].

ACE inhibitors are actively investigated for drug repurposing, both as single agents and in combination with other pharmacological compounds. In particular, combinations of ACE inhibitors with cytotoxic drugs have been proposed as potentially effective strategies in cancer therapy. It is known that some ACE inhibitors are capable of inhibiting matrix metalloproteinases (MMPs) in addition to ACE [20,21,22]. For example, captopril-mediated inhibition of MMP2 in gliosarcoma cells was shown to suppress cell migration, and in combination with the chemotherapeutic agent temozolomide, this approach increased survival in in vivo models, suggesting its potential utility in gliosarcoma treatment [22]. Notably, captopril in combination with chemotherapeutic agents is already being evaluated in clinical trials aimed at assessing its efficacy in oncological settings [23].

Using the proposed text-mining framework, we also extracted information on the potential application of ACE inhibitors in the treatment of neoplastic diseases other than gliosarcoma, in some cases accompanied by mechanistic insights underlying these effects. For example, captopril has been reported to exert a beneficial effect in colorectal cancer therapy, particularly by preventing liver metastasis [24,25]. Our analysis further revealed evidence that captopril may facilitate the delivery of gemcitabine in pancreatic cancer, where drug penetration is hindered by desmoplastic changes surrounding the tumor tissue, through inhibition of signaling pathways associated with tumor necrosis factor beta–SMAD family member 2 (TNF-β1–Smad2) [26]. In addition, captopril has been shown to reduce the risk of hepatocellular carcinoma development in the context of liver injury [27].

ACE inhibitors have also been investigated in the context of degenerative changes in internal organs. Captopril has been proposed as a preventive agent against renal fibrosis [28], while enalapril has been studied for the prevention of hypertrophic scarring when applied topically [29] and for the treatment of liver fibrosis [30]. These effects are attributed to the involvement of ACE inhibitors in pathways related to collagen synthesis and connective tissue formation.

Furthermore, the application of the proposed approach revealed that ACE inhibitors are being explored as potential therapeutic agents for depressive disorders [31,32]. The antidepressant effect of captopril has been hypothesized to involve bradykinin-dependent activation of the mechanistic target of rapamycin complex 1 (mTORC1) signaling pathway, as demonstrated in vivo [31]. In contrast, the effect of lisinopril has been associated with the ability of the renin–angiotensin system to modulate activity of the brain-derived neurotrophic factor/tropomyosin receptor kinase B (BDNF/TRKB) signaling pathway, which is implicated in the pathogenesis of depressive disorders [32].

Finally, we did not identify prominent examples of previously unrecognized adverse effects of ACE inhibitors that are not already documented in clinical guidelines or prescribing information. Nevertheless, well-known adverse effects of ACE inhibitors, such as dry cough, allergic reactions, and related conditions, were successfully identified. This observation further underscores the applicability of the proposed approach for extracting information on drug-associated adverse effects from biomedical texts.

### 3.3. Methodological Limitations

Several limitations of the proposed approach should be acknowledged. First, association extraction is performed at the sentence level using a predefined set of lexical patterns, which inherently constrains the range of relations that can be detected and may bias the results toward explicitly stated interactions. Mechanistic or clinical relationships described implicitly, across sentence boundaries, or using uncommon linguistic constructions are therefore likely to be underrepresented. Second, although entity normalization enables aggregation of evidence across publications, it remains susceptible to errors arising from abbreviation ambiguity and synonym variability, particularly for short or highly polysemous terms. Such cases may lead to artificial fragmentation or misassignment of entities in the aggregated networks and require post-extraction harmonization for precise biological interpretation. As a result, abbreviation recognition and disambiguation are only partially automated within the current implementation of the pipeline. Finally, the confidence score used in this study reflects a composite measure of textual and contextual features rather than a probabilistic estimate of biological validity; consequently, confidence thresholds should be interpreted as relative filters for prioritization rather than as absolute indicators of mechanistic certainty. An additional limitation of large-scale literature aggregation is related to the temporal instability of published evidence. It has been shown that findings reported at earlier stages of research are frequently revised, contradicted, or lose significance as additional data accumulate over time [33,34]. In this context, the limited temporal persistence observed for in vitro-only associations likely reflects their exploratory nature, whereas associations supported by in vivo and clinical studies tend to exhibit greater stability and recurrent confirmation across the literature. Accordingly, temporal recurrence was used in this study as an indirect indicator of evidential consolidation, partially mitigating the risk of overemphasizing repeatedly restated early findings. As illustrated in the Discussion, canonical mechanistic relations that dominate the extracted networks (e.g., ACE inhibition by ACE inhibitors) are repeatedly restated across publications [8,9,10,11,12,13,14,15,16], whereas low-frequency associations discussed in repurposing and non-cardiovascular contexts tend to originate from a limited number of studies and exhibit restricted temporal persistence [19,20,21,22,23,24,25,26,27,28,29,30,31,32]. This contrast reflects a literature-driven “echo” of well-established findings versus sparsely represented exploratory observations, consistent with previously described patterns of selective reporting and temporal instability of early results [33,34,35].

Text-mining-based aggregation relies on heterogeneous textual evidence rather than on well-defined experimental comparisons, which complicates the separation of meaningful associations from context-driven or incidental ones. As a result, some extracted relations may reflect non-specific contextual descriptions or coincidental mention patterns rather than robust biological effects, particularly at low levels of textual support. In the present framework, such associations tend to receive lower confidence scores due to limited contextual strength and sparse recurrence across independent sources. Confidence-based filtering therefore provides a practical mechanism for suppressing weakly supported relations, while more systematic strategies for characterizing background association patterns such as the use of reference networks derived from unrelated or randomly sampled drug classes represent an important direction for future methodological refinement.

Despite these limitations, the approach provides a scalable and transparent framework for consolidating heterogeneous textual evidence and for guiding targeted expert review of the extracted associations.

### 3.4. Added Value for Characterizing the Therapeutic Profile of ACE Inhibitors

Although the associations discussed above are grounded in previously published studies, the added value of the proposed text-mining and aggregation framework lies in its ability to systematically consolidate dispersed evidence and to highlight low-frequency, non-obvious links within a unified representation. For a well-established drug class such as ACE inhibitors, the mainstream therapeutic profile is expected to dominate the literature and is therefore readily recovered by most qualitative reviews. In contrast, less common combinations, repurposing directions, and mechanistically framed observations are typically scattered across heterogeneous publication types and therapeutic areas. By aggregating sentence-level evidence across the corpus and representing it in a structured form, the proposed approach enables efficient identification and prioritization of such low-frequency associations for targeted expert assessment and hypothesis generation.

In the co-prescription space, beyond the expected prevalence of standard cardiovascular combination regimens [18], the framework highlights rarer associations that intersect with metabolic or non-cardiovascular indications. A representative example is the reported benefit of combining lisinopril with empagliflozin in mouse models of diabetic nephropathy, where the combination outperformed monotherapy by slowing structural kidney damage [19]. Similarly, the extracted evidence points to repurposing-oriented combinations of ACE inhibitors with cytotoxic agents in oncology, supported by reports that some ACE inhibitors can inhibit matrix metalloproteinases in addition to ACE [20,21]. In particular, captopril-mediated inhibition of MMP2 has been linked to reduced gliosarcoma cell migration and improved survival when combined with temozolomide in vivo [22], with clinical evaluation of captopril-containing chemotherapeutic combinations also being reported [23]. These examples illustrate how the pipeline can surface clinically and mechanistically relevant combinations that are not dominant in the ACE inhibitor literature but may be of interest for repositioning-oriented analyses.

Beyond co-prescription, the approach also facilitates the retrieval of less central therapeutic themes and mechanistic hypotheses associated with ACE inhibitors across diverse disease contexts. In oncology, the extracted evidence includes reports of captopril in colorectal cancer with an emphasis on prevention of liver metastasis [24,25], mechanistic statements linking captopril to improved gemcitabine delivery in pancreatic cancer via pathways associated with TNF-β1–Smad2 [26], and observations suggesting a reduced risk of hepatocellular carcinoma under conditions of liver injury [27]. In fibrotic and degenerative conditions, the retrieved associations include proposals of captopril for prevention of renal fibrosis [28] and enalapril for topical prevention of hypertrophic scarring [29] and for liver fibrosis [30], consistent with connective-tissue–related pathways. Finally, the extracted evidence extends to neuropsychiatric indications, where ACE inhibitors have been discussed as potential agents for depressive disorders, including hypotheses involving bradykinin-dependent activation of mTORC1 for captopril [31] and modulation of BDNF/TRKB-related signaling for lisinopril [32]. Importantly, these findings should not be interpreted as novel pharmacological discoveries; rather, they demonstrate that the proposed framework can rapidly map the breadth of reported therapeutic and mechanistic contexts for a drug class and can support the generation of testable hypotheses by prioritizing low-frequency, literature-supported signals for deeper review.

## 4. Materials and Methods

In this study, we applied an automated text-mining pipeline to systematically extract and analyze associations involving angiotensin-converting enzyme inhibitors from the biomedical literature. The workflow comprised consecutive stages of literature retrieval, text preprocessing, named entity recognition, rule-based association extraction, entity normalization, and confidence assessment of the extracted relationships. All stages of the analysis were implemented in Python v 3.11.

A curated list of ACE inhibitors served as the basis for corpus construction and query formulation. Relevant publications were retrieved from PubMed using drug-specific queries combined with filters corresponding to different study types, including in vitro, in vivo, and clinical investigations. Titles and abstracts of the selected publications were automatically downloaded and processed, and each publication was associated with one or more study categories depending on the experimental context described.

Biomedical entities mentioned in the texts, including chemical compounds, genes/proteins, and diseases, were automatically recognized using a state-of-the-art neural network named entity recognition model. Associations between entities were subsequently extracted using a rule-based approach that relied on manually curated lexical patterns indicative of specific types of relationships. These patterns were grouped into several semantic categories and annotated with signal strength levels reflecting the expected reliability of the corresponding linguistic evidence.

To enable integration and comparison of extracted information, entities participating in the identified associations were normalized to external reference databases. Finally, individual mentions of associations were aggregated across publications, and a confidence score was assigned to each unique association based on the strength of the triggering pattern, positional proximity of entities within sentences, and the number of independent literature sources supporting the relationship.

### 4.1. Corpus Construction and Text Preprocessing

The corpus of publications was constructed using the PubMed database as a primary source of biomedical literature. A predefined list of angiotensin-converting enzyme inhibitors (ACE inhibitors) was used to formulate search queries. This list was obtained from the World Wide Approved Drugs (WWAD) database [36,37], which provides information on approved pharmaceutical substances and their therapeutic applications across multiple countries. The considered compounds included captopril, enalapril, lisinopril, enalaprilat, cilazapril, benazepril, fosinopril, quinapril, ramipril, delapril, racecadotril, perindopril, spirapril, moexipril, trandolapril, zofenopril, alacepril, imidapril, and temocapril.

For each compound, alternative names and commonly used synonyms were additionally included in the search queries to improve recall of relevant publications. Information on synonymous drug names was retrieved from the ChEMBL database [38], which integrates curated data on bioactive small molecules and their identifiers used in the biomedical literature.

To ensure coverage of different experimental contexts, three categories of studies were considered: in vitro, in vivo, and clinical investigations. For in vitro studies, queries combined drug names with MeSH terms related to cell-based experimental systems (e.g., “Cells”, “Cell Line”, “In Vitro Techniques”). In vivo studies were identified using MeSH terms associated with animal experiments (e.g., “Animals”) and corresponding textual markers. Clinical studies were retrieved based on publication types indicative of clinical research, including “Clinical Trial”, “Controlled Clinical Trial”, “Randomized Controlled Trial”, “Multicenter Study”, and related categories. Review articles, meta-analyses, editorials, letters, comments, and news items were explicitly excluded from all queries to focus the corpus on original experimental and clinical research.

Titles and abstracts of the identified publications, together with their metadata (PMID, publication year, journal, publication type), were automatically downloaded using Python scripts and the Bio.Entrez module. Each publication could be associated with more than one study category if multiple experimental contexts were described.

The collected texts were subjected to standard preprocessing procedures prior to further analysis. Titles and abstracts were concatenated and cleaned by removing non-informative characters and normalizing whitespace. The resulting text was then segmented into individual sentences using rule-based sentence boundary detection. Sentence-level representation was preserved throughout subsequent stages of the analysis, allowing each extracted association to be traced back to a specific sentence and publication.

### 4.2. Named Entity Recognition

Automatic recognition of biomedical entities was performed using a neural network–based named entity recognition (NER) model. Specifically, we employed the HunFlair2 framework, a hybrid NER system that leverages contextualized language representations derived from pretrained transformer models in combination with sequence labeling techniques, and is trained on multiple large-scale biomedical corpora for robust recognition of domain-specific entities [39]. The model was applied to the preprocessed texts at the sentence level, enabling precise localization of entity mentions within individual sentences.

Three types of biomedical entities were considered in this study: chemical compounds, genes/proteins, and diseases. Along with the recognized entity text, the start and end character positions within the sentence were retained for downstream analysis.

Entity recognition was applied uniformly across all collected sentences without prior filtering. However, only entities participating in candidate associations identified at later stages of the pipeline were subjected to further processing, such as normalization to external databases. This strategy reduced unnecessary computational overhead while preserving full coverage of potentially relevant entity mentions.

### 4.3. Rule-Based Association Extraction

Extraction of associations between biomedical entities was performed using a rule-based approach operating at the sentence level. This approach was chosen due to its interpretability and the possibility of control over the types of relations being extracted [6,40].

A curated set of lexical patterns (hereafter referred to as pattern phrases) was used to identify relational contexts within sentences. These pattern phrases were derived through manual analysis of a representative subset of the collected corpus and subsequently expanded to include common morphological and lexical variants (e.g., different verb forms and derivational variants). Each pattern phrase was assigned to one of three predefined semantic groups corresponding to the type of association being extracted:

(1) mechanisms of action involving chemical compounds and genes or proteins,
(2) effects of chemical compounds on diseases, and
(3) co-prescription or co-administration of chemical compounds.

For each semantic group, pattern phrases were additionally categorized by their indicative strength (strong, medium, or weak), reflecting the degree to which their presence in a sentence suggests a meaningful association. This categorization was later taken into account during confidence scoring of the extracted associations.

Association extraction was based on the co-occurrence of three elements within a single sentence: (i) a mention of an angiotensin-converting enzyme inhibitor from a predefined list, (ii) a second biomedical entity (gene or protein, disease, or another chemical compound, depending on the association group), and (iii) a pattern phrase belonging to the corresponding semantic group. Only sentences containing all three elements were considered candidates for association extraction. Chemical compound mentions not corresponding to ACE inhibitors were retained only when participating as the second entity in co-prescription associations.

To account for negated statements, a negation detection procedure was applied for associations belonging to the first two semantic groups (mechanisms and effects). The presence of negation cues (e.g., no, not, without, lack of, fail to) within a predefined window of five tokens before or after the pattern phrase resulted in the corresponding association being marked as negative. Such associations were retained in the dataset but annotated and treated separately in subsequent analyses.

For each candidate association, the relative distance between the pattern phrase and the participating entities was taken into account. The distance was defined as the minimum number of tokens separating the pattern phrase from each entity mention within the sentence. Associations were scored based on this distance, with shorter distances corresponding to higher confidence values, reflecting a stronger contextual linkage between the entities and the triggering phrase. For each sentence, only associations with the minimal distance-based score were retained, thereby reducing noise from loosely connected co-occurrences and prioritizing the most contextually grounded relations.

### 4.4. Entity Normalization

To reduce lexical variability and enable consistent aggregation of extracted associations, all biomedical entity mentions participating in associations were normalized to unique identifiers from external reference databases. Normalization was performed only for entities involved in extracted associations, thereby avoiding unnecessary processing of entity mentions that did not contribute to relational evidence.

Chemical compound mentions corresponding to angiotensin-converting enzyme inhibitors and co-prescribed drugs were normalized to PubChem [41] compound identifiers using programmatic queries based on the recognized entity names. This approach enabled unambiguous identification of chemical substances and enabled discrimination between angiotensin-converting enzyme inhibitors and other chemical compounds mentioned in the texts, ensuring that the extracted associations remained focused on the target drug class.

Mentions of genes and proteins were normalized to UniProt entries [42]. In cases where entity mentions were ambiguous with respect to organism origin, priority was given to human proteins when sufficient contextual information was available. When organism information could not be reliably inferred from the sentence or associated metadata, normalization was performed conservatively, and the corresponding organism annotation was retained where possible.

Disease mentions were normalized to terms from the Human Disease Ontology [43], providing a structured and hierarchically organized representation of disease entities. This normalization step allowed integration of disease-related associations across synonymous or partially overlapping textual mentions.

Associations for which at least one participating entity could not be confidently normalized were retained in the dataset but flagged accordingly and excluded from analyses requiring fully normalized entity pairs. This strategy ensured transparency of the normalization process while preserving potentially informative associations for further manual inspection.

### 4.5. Aggregation and Confidence Scoring of Associations

After entity normalization, extracted sentence-level associations were aggregated across the entire corpus to obtain a non-redundant set of relations. Associations were considered identical if they involved the same normalized entity pair, belonged to the same semantic group, and were characterized by the same relation type. This aggregation step ensured that each unique association was represented only once in the final dataset, while preserving information about all supporting textual evidence.

For each aggregated association, all PubMed identifiers from which it was extracted were retained, along with the corresponding study types (in vitro, in vivo, and/or clinical). If an association was supported by evidence originating from multiple experimental contexts, all relevant study types were recorded. This representation enabled traceability of each association to its original sources and facilitated assessment of evidence diversity.

To estimate the reliability of the extracted associations, a composite confidence score was calculated by integrating multiple complementary components reflecting both linguistic and corpus-level evidence. At the sentence level, each candidate association was assigned a local score based on two factors: (i) the indicative strength of the triggering pattern phrase and (ii) the relative distance between the pattern phrase and the participating entities within the sentence.

Indicative strength was modeled as a discrete weight $w_{p}$, assigned according to the category of the pattern phrase: $w_{p}=3$ for strong patterns, $w_{p}=2$ for medium patterns, $w_{p}=1$ for weak patterns.

Contextual proximity was captured by a distance-based component d, defined as the minimum number of tokens separating the pattern phrase from each participating entity mention. Shorter distances were assumed to reflect stronger contextual linkage. The sentence-level score $S_{sent}$ for an association candidate was therefore defined as:

$$S_{sent}=\frac{w_{p}}{1+d}$$

For each sentence, only the candidate association with the highest sentence-level score was retained, thereby reducing noise from loosely connected co-occurrences and prioritizing the most contextually grounded relations.

At the corpus level, aggregated associations were further weighted by the number of independent publications in which they were observed. Let $N_{pub}$ denote the number of unique PMIDs supporting a given association. The final confidence score $S_{conf}$ was computed as:

$$S_{conf}=\sum_{i=1}^{N_{pub}}S_{sent}^{\left(i\right)}$$

where $S_{sent}^{\left(i\right)}$ represents the sentence-level score obtained from the *i*-th publication. This formulation naturally increases the confidence of associations repeatedly observed across independent sources, while still allowing rare or emerging associations to be retained with lower scores.

Associations marked as negated during sentence-level extraction were aggregated separately and assigned confidence scores using the same procedure. Although such associations do not constitute positive evidence of a relationship, their explicit representation provides important contextual information, including experimentally tested but unsupported or condition-dependent effects. Differences in confidence scores between positive and negative associations may be informative when selecting the most contextually appropriate interpretation of a relation. However, positive and negative associations were not combined into a single confidence score, as they represent different types of experimental statements. Aggregating them into a unified measure could introduce bias, given that the underlying sources do not allow objective weighting of evidence supporting or refuting a relationship. Therefore, positive and negative associations were evaluated separately to preserve interpretability and avoid distortion of confidence estimates.

Overall, this aggregation and scoring strategy integrates linguistic evidence, contextual coherence, and reproducibility across publications into a single quantitative measure. The resulting confidence score enables prioritization of associations supported by strong pattern phrases, close contextual proximity, and multiple independent sources, while maintaining transparency and interpretability of the extraction process.

## 5. Conclusions

This study demonstrates the applicability of an integrative text-mining approach for the systematic analysis of pharmacological mechanisms, therapeutic effects, and co-prescription patterns of ACE inhibitors. Using an in-house developed rule-based extraction framework combined with a composite confidence score, we aggregated sentence-level associations from a large biomedical corpus and represented them in a structured and interpretable form.

The results confirm that the dominant mechanistic and therapeutic profiles of ACE inhibitors reported in the literature are centered on canonical inhibition of the angiotensin-converting enzyme and established antihypertensive combination regimens. At the same time, the proposed approach enabled the identification and prioritization of less frequent, literature-supported associations that are typically scattered across publications. These include co-prescription patterns involving metabolic agents (e.g., combinations of ACE inhibitors with SGLT2 inhibitors), repurposing-oriented associations in oncology linked to matrix metalloproteinase inhibition, and mechanistically framed evidence for potential roles of ACE inhibitors in fibrotic and neuropsychiatric conditions. Although these findings are not novel discoveries per se, their aggregation highlights non-obvious therapeutic contexts and supports hypothesis generation for further experimental or clinical evaluation.

The analysis also illustrates the importance of confidence-based filtering and polarity annotation for interpreting extracted associations, particularly in well-studied pharmacological systems where contradictory or context-dependent statements are common. Network representations proved useful for distinguishing canonical, system-level, and low-frequency associations and for revealing normalization-related artifacts that warrant post-processing harmonization.

Several limitations of the approach should be noted. Association extraction is limited to sentence-level patterns and may miss relations expressed implicitly or across sentence boundaries. Entity normalization remains sensitive to abbreviation ambiguity and synonym variability, which can affect downstream aggregation. In addition, the confidence score reflects a relative, composite measure of textual support rather than a probabilistic estimate of biological validity and should therefore be used for prioritization rather than definitive inference.

Overall, the proposed framework provides a scalable and transparent method for consolidating heterogeneous textual evidence on drug mechanisms and therapeutic profiles. It can be readily extended to other drug classes and biomedical domains, where it may support literature-driven hypothesis generation, drug repurposing studies, and computer-aided drug design.

## Figures and Tables

**Table 1:** Distribution of ACE inhibitor-related texts by study type.

Drug	Absolute Values				Rates			
	IVT	IVV	C	Total	% IVT	% IVV	% C	% of Drug Texts
Enalapril	1733	4177	4725	10,635	16.30	39.28	44.43	45.66
Captopril	2405	5438	1742	9585	25.09	56.73	18.17	41.15
Benazepril	737	1297	2057	4091	18.02	31.70	50.28	17.56
Ramipril	357	792	746	1895	18.84	41.79	39.37	8.14
Lisinopril	408	719	758	1885	21.64	38.14	40.21	8.09
Perindopril	248	589	675	1512	16.40	38.96	44.64	6.49
Enalaprilat	410	771	168	1349	30.39	57.15	12.45	5.79
Quinapril	121	284	245	650	18.62	43.69	37.69	2.79
Trandolapril	68	162	253	483	14.08	33.54	52.38	2.07
Cilazapril	77	200	197	474	16.24	42.19	41.56	2.03
Fosinopril	89	170	175	434	20.51	39.17	40.32	1.86
Imidapril	52	142	61	255	20.39	55.69	23.92	1.09
Temocapril	51	94	32	177	28.81	53.11	18.08	0.76
Zofenopril	48	73	45	166	28.92	43.98	27.11	0.71
Racecadotril	33	85	43	161	20.50	52.80	26.71	0.69
Delapril	20	58	44	122	16.39	47.54	36.07	0.52
Spirapril	10	35	63	108	9.26	32.41	58.33	0.46
Moexipril	14	26	32	72	19.44	36.11	44.44	0.31
Alacepril	20	38	11	69	28.99	55.07	15.94	0.30
Total texts	23,294							

IVT—in vitro, IVV—in vivo, C—clinical.

**Table 2:** Distribution and structural characteristics of rule-based pattern groups used for association extraction.

Group	N SG	N PT	Mean PT per SG	Median PT per SG	N Strong	N Medium	N Weak
Mechanisms	42	248	5.90	6	9	23	10
Effects	33	167	5.06	6	6	14	13
Co-administration	3	13	4.43	3	1	1	1

SG—pattern subgroups; PT—lexical pattern phrases. N SG—number of subgroups; N PT—total number of pattern phrases; Mean/Median PT per SG—mean/median number of pattern phrases per subgroup; N strong/medium/weak—numbers of subgroups with corresponding indicative strength.

**Table 3:** Top 20 relation types among aggregated associations extracted from the literature.

Group	Subgroup	N Associations
Mechanisms	Inhibit	2086
Effects	Effect	1361
Effects	Treatment	1247
Effects	Patient	1133
Mechanisms	Increase	856
Effects	Reduce	783
Mechanisms	Decrease	759
Mechanisms	Reduce	750
Effects	Induce	730
Co-prescription	Cooccurrence	685
Mechanisms	Antagonize	637
Co-prescription	Coadministered_with	629
Effects	Prevent	573
Effects	Improve	483
Effects	Decrease	482
Mechanisms	Attenuate	421
Mechanisms	Block	418
Mechanisms	Induce	407
Effects	Therapy	406
Effects	Receive	399

**Table 4:** Distribution of aggregated associations by signal strength across semantic groups.

PatternGroupName	SignalStrength_Best	N Associations
Co-prescription	strong	629
Co-prescription	medium	10
Co-prescription	weak	685
Effects	strong	2228
Effects	medium	4240
Effects	weak	3944
Mechanisms	strong	4275
Mechanisms	medium	5514
Mechanisms	weak	870

**Table 5:** Number of aggregated associations retained at different confidence score thresholds.

Confidence Score Threshold (≥)	Mechanisms	Effects	Co-Prescription
0.1	479	314	128
0.15	252	117	34
0.2	163	52	14
0.3	93	10	0
0.4	60	2	0
0.5	46	0	0
0.6	33	0	0
0.7	27	0	0

Cells shaded in gray indicate associations that meet the confidence score threshold applied for interaction network visualization.

## Data Availability

The original contributions presented in this study are included in the article/Supplementary Materials. Further inquiries can be directed to the corresponding authors.

## Supplementary Materials

The following supporting information can be downloaded at https://www.mdpi.com/article/10.3390/ijms27042044/s1.

## Abbreviations

The following abbreviations are used in this manuscript:

ACE	Angiotensin-Converting Enzyme
ACEi	Angiotensin-Converting Enzyme Inhibitor
Ang II	Angiotensin II
DOID	Human Disease Ontology Identifier
MMP	Matrix Metalloproteinase
NER	Named Entity Recognition
RAS	Renin–Angiotensin System
